# Supplementary material for: EnzML: multi-label prediction of enzyme classes using InterPro signatures
Source: BMC Bioinformatics. 2012 Apr 25;13:61. doi: 10.1186/1471-2105-13-61 (PMC3483700; doi:10.1186/1471-2105-13-61)
Supplement: Addtional file 5 — The Java code to format the data files, evaluate and predict. The file enzml_java_code.tar.gz contains the Java code used to format database data to ARFF and XML formats, to execute cross and train-test (jackknife) evaluations and to record evaluation results to database. More information is included in the readme.txt file and the Javadoc files. The code can be used with a MySQL database. To use a different database software, other JDBC drivers might be required. [file 1471-2105-13-61-S5.gz › java_code/utils/doc/cern/jet/random/AbstractDistribution.html]

AbstractDistribution


---


|  |  |  |
| --- | --- | --- |
| **Overview** \| **Package** \| **Class** \| **Use** \| **Tree** \| **Deprecated** \| **Index** \| **Help** | |  |
| **PREV CLASS**   **NEXT CLASS** | **FRAMES**    **NO FRAMES**     **All Classes** |  |
| SUMMARY: NESTED \| FIELD \| CONSTR \| METHOD | DETAIL: FIELD \| CONSTR \| METHOD |  |


---


## cern.jet.random Class AbstractDistribution

```
java.lang.Object
  cern.colt.PersistentObject
      cern.jet.random.AbstractDistribution
```

**All Implemented Interfaces:**: DoubleFunction, IntFunction, java.io.Serializable, java.lang.Cloneable

**Direct Known Subclasses:**: AbstractContinousDistribution

---

``` public abstract class AbstractDistribution extends PersistentObject implements DoubleFunction, IntFunction ```

Abstract base class for all random distributions.
A subclass of this class need to override method nextDouble() and,
in rare cases, also nextInt().

Currently all subclasses use a uniform pseudo-random number generation engine
and transform its results to the target distribution. Thus, they expect such
a uniform engine upon instance construction.

`MersenneTwister` is recommended as uniform
pseudo-random number generation engine, since it is very strong and at the
same time quick. `makeDefaultGenerator()` will conveniently construct
and return such a magic thing. You can also, for example, use
`cern.jet.random.engine.DRand`, a quicker (but much weaker) uniform
random number generation engine. Of course, you can also use other strong
uniform random number generation engines. Paul Houle's RngPack package
contains some such beasts; `Ranecu`,
`Ranecu` and
`Ranlux` are strong well analyzed
research grade uniform pseudo-random number generators with known periods.
More info on RngPack can be found  here.

**Ressources on the Web:**

Check the Web version of the CERN Data Analysis Briefbook . This will clarify the definitions of most distributions. Also consult the StatSoft Electronic Textbook - the definite web book. **Other useful ressources:** Another site and yet another site describing the definitions of several distributions. You may want to check out a Glossary of Statistical Terms. The GNU Scientific Library contains an extensive (but hardly readable) list of definition of distributions. Use this Web interface to plot all sort of distributions. Even more ressources: Internet glossary of Statistical Terms, a text book, another text book. Finally, a good link list Statistics on the Web. **Version:** : 1.0, 09/24/99 **Author:** : wolfgang.hoschek@cern.ch **See Also:**: `cern.jet.random.engine`, `edu.cornell.lassp.houle.RngPack`, `cern.jet.random.engine.Benchmark`, `cern.jet.random.Benchmark`, Serialized Form --- | **Method Summary** | | | --- | --- | | `double` | `apply(double dummy)`           Equivalent to nextDouble(). | | `int` | `apply(int dummy)`           Equivalent to nextInt(). | | `java.lang.Object` | `clone()`           Returns a deep copy of the receiver; the copy will produce identical sequences. | | `static RandomElement` | `makeDefaultGenerator()`           Constructs and returns a new uniform random number generation engine seeded with the current time. | | `abstract  double` | `nextDouble()`           Returns a random number from the distribution. | | `int` | `nextInt()`           Returns a random number from the distribution; returns (int) Math.round(nextDouble()). | **Methods inherited from class java.lang.Object** `equals, getClass, hashCode, notify, notifyAll, toString, wait, wait, wait` **Method Detail** ### apply ``` public double apply(double dummy) ``` : Equivalent to nextDouble(). This has the effect that distributions can now be used as function objects, returning a random number upon function evaluation. : **Specified by:**: `apply` in interface `DoubleFunction` : **Parameters:**: `dummy` - argument passed to the function. **Returns:**: the result of the function. --- ### apply ``` public int apply(int dummy) ``` : Equivalent to nextInt(). This has the effect that distributions can now be used as function objects, returning a random number upon function evaluation. : **Specified by:**: `apply` in interface `IntFunction` : **Parameters:**: `dummy` - argument passed to the function. **Returns:**: the result of the function. --- ### clone ``` public java.lang.Object clone() ``` : Returns a deep copy of the receiver; the copy will produce identical sequences. After this call has returned, the copy and the receiver have equal but separate state. : **Overrides:**: `clone` in class `PersistentObject` : **Returns:**: a copy of the receiver. --- ### nextDouble ``` public abstract double nextDouble() ``` : Returns a random number from the distribution. --- ### nextInt ``` public int nextInt() ``` : Returns a random number from the distribution; returns (int) Math.round(nextDouble()). Override this method if necessary. --- ### makeDefaultGenerator ``` public static RandomElement makeDefaultGenerator() ``` : Constructs and returns a new uniform random number generation engine seeded with the current time. Currently this is `MersenneTwister`. --- | | | | | --- | --- | --- | | **Overview** \| **Package** \| **Class** \| **Use** \| **Tree** \| **Deprecated** \| **Index** \| **Help** | | | | **PREV CLASS**   **NEXT CLASS** | **FRAMES**    **NO FRAMES**     **All Classes** | | | SUMMARY: NESTED \| FIELD \| CONSTR \| METHOD | DETAIL: FIELD \| CONSTR \| METHOD | | ---
